# A Convolutional Neural Network with Multifrequency and Structural Similarity Loss Functions for Electromagnetic Imaging

**DOI:** 10.3390/s24154994

**Published:** 2024-08-01

**Authors:** Chien-Ching Chiu, Che-Yu Lin, Yu-Jen Chi, Hsiu-Hui Hsu, Po-Hsiang Chen, Hao Jiang

**Affiliations:** 1 Department of Electrical and Computer and Engineering, Tamkang University, New Taipei City 251301, Taiwan; 811440030@o365.tku.edu.tw (C.-Y.L.); yjchi@mail.tku.edu.tw (Y.-J.C.); 409490074@gms.tku.edu.tw (H.-H.H.); 810440031@gms.tku.edu.tw (P.-H.C.); 2 School of Engineering, San Francisco State University, San Francisco, CA 94117-1080, USA; jianghao@sfsu.edu

**Keywords:** electromagnetic imaging, artificial intelligence, anisotropic objects, back-propagation scheme, loss function, convolutional neural network

## Abstract

In this paper, artificial intelligence (AI) technology is applied to the electromagnetic imaging of anisotropic objects. Advances in magnetic anomaly sensing systems and electromagnetic imaging use electromagnetic principles to detect and characterize subsurface or hidden objects. We use measured multifrequency scattered fields to calculate the initial dielectric constant distribution of anisotropic objects through the backpropagation scheme (BPS). Later, the estimated multifrequency permittivity distribution is input to a convolutional neural network (CNN) for the adaptive moment estimation (ADAM) method to reconstruct a more accurate image. In the meantime, we also improve the definition of loss function in the CNN. Numerical results show that the improved loss function unifying the structural similarity index measure (SSIM) and root mean square error (RMSE) can effectively enhance image quality. In our simulation environment, noise interference is considered for both TE (transverse electric) and TM (transverse magnetic) waves to reconstruct anisotropic scatterers. Lastly, we conclude that multifrequency reconstructions are more stable and precise than single-frequency reconstructions.

## 1. Introduction

Advances in magnetic anomaly sensing systems and electromagnetic imaging use electromagnetic principles to detect and characterize subsurface or hidden objects. Magnetic anomaly sensing systems primarily detect changes in the earth’s magnetic field caused by ferromagnetic objects or anomalies. Electromagnetic imaging uses electromagnetic waves to create detailed images of the interior of an object, such as dielectric constant and conductivity. Combining these two technologies will result in a more comprehensive sensing and imaging solution to improve the accuracy and depth of subsurface investigations, consequently serving as a powerful tool for applications such as environmental monitoring, safety inspections, and medical diagnostics. Artificial intelligence (AI) technology is used in various fields and has flourished in recent years [1,2,3,4,5,6,7,8]. Overall, electromagnetic imaging technology by AI plays an important role in modern science and engineering and will continue to be developed and applied in the future.

In the current academic research, the following two approaches are used to solve electromagnetic imaging: algorithms [9,10,11,12,13] and AI [14,15,16,17,18]. In 2019, Zhou proposed that a non-decimated wavelet transform based on an iterative method with adaptive thresholding for the compressed sensing method (NDW-IMATCS) combined with the dual-mesh method could achieve fast, accurate and stable reconstruction of nonsparse objects [9]. In 2020, Wei proposed a novel computational approach to solve the forward scattering problem, ensuring accurate convergence of the total field by incorporating high-order components. [10]. To solve the 3-D inverse scattering problem effectively, in 2021, Zhao et al. introduced an enhanced subspace-regularized distorted Born iterative method, utilizing a multilevel Green’s function interpolation approach to expedite the computation of the forward problem. [11]. In 2022, Saraskanroud proposed two hybrid time domain (TD) and frequency domain (FD) microwave imaging schemes. This hybrid method combined the discontinuous Galerkin method implementation of the TD forward–backward time stepping (FBTS) algorithm and FD contrast source inversion (CSI) or gauss–newton inversion (GNI) to shorten the computing time of the quantitative TD imaging algorithm and improve the image resolution of the quantitative FD imaging algorithm. The results also showed that low-resolution FD prior information could improve TD convergence [12]. In 2023, Sun proposed a fast algorithm for the cross-correlation contrast source inversion (CC-CSI) method. By assuming a uniform background medium, the most time-consuming part of the CC-CSI method in the linear equation system was solved. And the 3-D inversion of transverse magnetic (TM) and transverse electric (TE) was effectively realized through multiple fast Fourier transforms and inverse fast Fourier transforms, respectively. Furthermore, this rapid approach applied to all inversion methods concerning the direct calculation of electric fields from contrasting sources in homogeneous background media [13].

AI is another option that has been commonly used in recent years to generate electromagnetic imaging [14,15,16,17,18]. In 2019, Wei proposed a convolutional neural network (CNN) technique to solve the full-wave inverse scattering problem (ISP). He compared three U-Net CNN-based training schemes, namely, backpropagation, dominant current scheme, and direct inversion. The results showed that the proposed dominant current scheme outperformed the other two schemes in terms of accuracy and timeliness. It could resolve a typical ISP within 1 s [14]. In 2020, Xiao proposed a 3-D electromagnetic inversion method based on Born approximation and a CNN to reconstruct non-uniform scatterers with complex shapes. The result showed that 3-D U-Net outperformed the traditional variational Born iteration method in both accuracy and efficiency [15]. In 2021, Ma presented a learning-based non-iterative approach to solve the ISP using the generative adversarial network (GAN) pix2pix. The forward-induced current learning method (FICLM) with direct sum of dielectric constant contrast and Born-type induced currents had the best computational accuracy and generalization capability. Compared with other types of neural networks, the adversarial framework in pix2pix provided FICLM superior performance in dealing with complex scatterers [16]. To bridge the gap between traditional model-based approaches and data-driven deep learning schemes, in 2022, Liu proposed a physical model-inspired deep unrolling network (PM-Net) to solve nonlinear ISP. Compared with traditional iterative methods, this mechanism, which learned fewer parameters, was comparable or even better than subspace-based optimization methods under high-noise environments [17]. In 2023, Wang investigated multiple-space deep learning schemes (MSDLSs) combining frequency-space and real-space processing. Through the complementary feature between the serial and parallel MSDLSs, dynamic interaction among multiple-space information could be achieved in both the training and testing stages [18].

Electromagnetic imaging is an imaging technique that applies low-frequency, high-frequency, and even multifrequency electromagnetic waves. Low-frequency electromagnetic waves have higher penetration and higher resolution because of their longer wavelengths. However, they easily interfere with other electromagnetic waves. High-frequency electromagnetic waves are fast and directional. They can be focused on a small area to achieve high-precision imaging. Nevertheless, their shorter wavelengths cause poorer penetration that restricts the imaging range. Multifrequency electromagnetic imaging emerged to overcome the defects of low- and high-frequency electromagnetic waves [19,20,21,22,23,24]. In 2020, Sangwoo proposed a MultiFrequency Direct Sampling Method (DSM) to solve the limited-aperture inverse scattering problem [19]. In 2021, Zhang proposed a subspace-based hierarchical optimization method that combined a subspace-based optimization method with a hierarchical strategy using multifrequency data to reconstruct 2-D uniaxial anisotropic scatterers. This method could reconstruct images efficiently [20]. In 2021, Li proposed a CNN that introduced a multi-channel scheme to solve the multifrequency ISP. The CNN inversion method could achieve acceptable quality results rapidly. The proposal also highlighted that the multifrequency CNN worked well in high contrast problems or more complex cases, as well as different frequency bands [21]. In 2022, Xie proposed a modified major current coefficient method combining the major current coefficients method, the hierarchical scheme, generalized Tikhonov regularization, and a frequency rejection mechanism to solve the ISP using multiple-frequency data. This method improved reconstruction performance within a limited view [22]. In 2022, Park considered a multifrequency DSM for fast recognition of linear perfectly conducting cracks with small lengths from measured far-field pattern data [23]. In 2023, Zhang inputted single-frequency EM scattering field information into a complex-valued deep residual convolutional neural network to predict multifrequency EM scattering fields. The resulting multifrequency EM scattering field “image” was then input to a new complex-valued convolutional encoder–decoder structure to regenerate target scatterers. This approach solved the ISP accurately and efficiently [24].

In most of the studies, TM waves were widely probed in various arenas. As TM waves are purely scalar, they are simpler for reconstruction, whereas TE waves have correlated x and y vectors that may interfere mutually, making them more complicated to process, especially when dealing with uniaxial objects [25,26,27,28]. In 2020, Wang proposed using the biconjugate gradient stabilized method and fast Fourier transform in the forward model to simulate the electromagnetic scattering of arbitrary anisotropic scatterers embedded in a layered arbitrary anisotropic background medium. Twelve dielectric parameters for each cell of the arbitrary anisotropic scatterer were reconstructed by VBIM-SCC-SCS. The shape of the scatterer as well as the anisotropic model parameters were satisfactorily reconstructed [25]. In 2022, Ye presented an efficient, accurate, and real-time inverse algorithm based on the super-resolution generative adversarial network for quantitative imaging of 2-D biaxial anisotropic scatterers. With this approach, image quality and resolution were greatly improved, and the computation time was also significantly reduced [26]. In 2022, Chiu proposed using a deep neural network to reconstruct the permittivity of uniaxial scatterers more efficiently. The results showed that the reconstructed permittivity performance by the dominant current scheme (DCS) was better than the backpropagation scheme (BPS) [27]. In 2023, Chiu proposed combining AI with a modified contrast scheme (MCS) technique to reconstruct microwave imaging of uniaxial objects. The results revealed that for a small dielectric constant, the performance for MCS and DCS was almost the same. The reconstruction for MCS turned out to be better when the dielectric constant became higher [28].

Our contributions are as follows:

(1) In this paper, we demonstrate a novel deep learning scheme for reconstructing anisotropic objects. In the neural network, we use a traditional U-Net with multi-loss to reconstruct the material, shape, and size of a dielectric object. Past studies have used RMSE as a reconstruction metric for neural networks. In this paper, we use SSIM and RMSE as reconstruction metrics for neural networks. The numerical results show that using SSIM and RMSE as the reconstruction metrics of the neural network can effectively improve the quality of electromagnetic images.
(2) Similar studies in the past, such as reference [21], only reconstructed EM images in the TM case. The advantage of this paper is that we successfully utilize multifrequency techniques to reconstruct anisotropic objects. In addition, we analyze the effects of different dielectric coefficient distributions and noise on the reconstruction of EM images by multifrequency techniques. Lastly, we also validate the efficacy of our proposed method with a research dataset.

We present the direct formulas in Section 2. The neural network architecture and loss function are described in Section 3. Section 4 presents an analysis of the numerical results of different case and research dataset. The conclusions are given in Section 5.

## 2. Theory Formulation

### 2.1. Direct Problem

**Figure 1:**
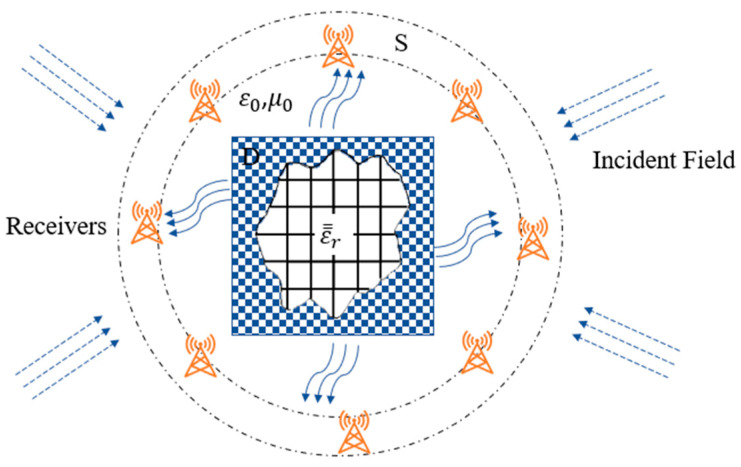
Simulation environment.

We assume an anisotropic object (scatterer) is immobilized in a closed surface S in free space with relative permittivity tensors ${\overset{̿}{\varepsilon }}_{r}$ and magnetic conductivity $\mu_{0}$, as shown in Figure 1. Here, a two-dimensional scenario is evaluated, namely, the material of the scatterer only changes with the x, y coordinates. The diagonal matrix of the Cartesian coordinate system can be used to define the dielectric constant distribution of the anisotropic object. We denote the dielectric constant along the z axis as $\varepsilon_{1}$ and along the x and y axes as $\varepsilon_{2}$ and $\varepsilon_{3},$ as shown in (1). Among them, $\varepsilon_{1}\left(x,y\right),\varepsilon_{2}\left(x,y\right),$ and $\varepsilon_{3}\left(x,y\right)$ are generally complex.

(1) $${{\overset{̿}{\varepsilon }}_{r}=\left[\begin{array}{ccc} \varepsilon_{2}\left(x,y\right) & 0 & 0\\ 0 & \varepsilon_{3}\left(x,y\right) & 0\\ 0 & 0 & \varepsilon_{1}\left(x,y\right) \end{array}\right]}_{xyz}$$

First, the incident field ${\overset{⃑}{E}}^{i}\left(x,y\right)$ with $e^{j\omega t}$ is regarded as time-dependent harmonics, and the $E_{z}^{i}\left(x,y\right)$ function is the magnitude of the incident field. Since the material properties of the scatterer are assumed to be independent of the z-direction, only TM-polarized scattered waves will be generated when irradiating TM-polarized waves (with the incident field in the z-direction). We irradiate TE and TM waves separately to reconstruct the dielectric coefficient of the anisotropic objects for comparison.

For the direct problem, we discretize the scatterer’s surface into many small enough regions, where the dielectric coefficient and electric field in each region are treated as constants. Let $\varepsilon_{1n}$, $\varepsilon_{2n}$, and $\varepsilon_{3n}$ represent the dielectric coefficients of the n-th region in the *z*-, *x*-, and *y*-directions, respectively. Then, (3)–(10) are solved using the method of moments and expanded with pulse basis functions. The Dirac delta function is eventually tested.

#### 2.1.1. TM (Transverse Magnetic) Waves

TM electromagnetic waves are irradiated in the region S. Equation (2) is the incident field $E_{z}^{i}(x,y)$. $k_{0}^{\;}$ is the free-space wave number and ξ is the incident angle.

(2) $${\bar{E}}^{i}\left(x,y\right)={E_{z}^{i}\left(x,y\right)\hat{z}}^{\;}=e^{-jk_{0}(x\cos\xi +y\sin\xi )}\hat{z}$$

Since the incident z-direction waves can only generate the z component scattered field, the total electric field and scattered field can be written as (3) and (4), respectively.

(3) $$\begin{array}{c} E_{z}^{\;}\left(x,y\right)=\int_{s}G\left(x,y;x^{\prime },y^{\prime }\right)\left(\varepsilon_{1}\left(x^{\prime },y^{\prime }\right)-1\right)E_{z}^{\;}\left(x^{\prime },y^{\prime }\right)ds^{\prime }\\ +E_{z}^{i}\left(x^{\prime },y^{\prime }\right),\;\left(x,y\right),\left(x^{\prime },y^{\prime }\right)\in S \end{array}$$

(4) $$E_{z}^{s}\left(x,y\right)=\int_{s}^{\;}G\left(x,y;x\prime ,y\prime \right)\left(\varepsilon_{1}\left(x\prime ,y\prime \right)-1\right)E_{z}^{\;}\left(x\prime ,y\prime \right)ds^{\prime },\;\overset{⃑}{r}\notin S,\;\overset{⃑}{r}\prime \in S$$

where $G\left(x,y;x\prime ,y\prime \right)=-\frac{jk_{0}^{2}}{4}H_{0}^{\left(2\right)}\left(k_{0}\left|\left(x,y)-(x\prime ,y\prime \right)\right|\right)$ is Green’s function of two-dimensional free space, and $H_{0}^{\left(2\right)}$ is the zero-order Hankel function of the second kind. Then, (3) and (4) can be converted into matrix equations as shown in (5) and (6), respectively,

(5) $$-\left(E_{z}^{i}\right)=\left(\left[G_{1}\right]\left[\tau_{z}\right]-\left[I\right]\right)\left(E_{z}^{\;}\right)$$

(6) $$\left(E_{z}^{s}\right)=\left[G_{2}\right]\left[\tau_{z}\right]\left(E_{z}^{\;}\right)$$

and the Green’s function matrices are as shown in (7) and (8)

(7) $${\left(G_{1}\right)}_{mn}=\left\{\begin{array}{c} -\frac{j\pi k_{0}a_{n}}{2}J_{1}\left(k_{0}a_{n}\right){H_{0}}^{\left(2\right)}\left(k_{0}\rho_{mn}\right),\;m\neq n\\ -\frac{j}{2}\left[{\pi k}_{0}a_{n}{H_{1}}^{\left(2\right)}\left(k_{0}a_{n}\right)-2j\right],\;m=n \end{array}\right.$$

(8) $${\left(G_{2}\right)}_{mn}=-\frac{j\pi k_{0}a_{n}}{2}J_{1}\left(k_{0}a_{n}\right){H_{0}}^{\left(2\right)}\left(k_{0}\;\rho_{mn}\right)$$

where $\left(E_{z}^{\;}\right)$ represents the total electric field column vectors of the N-element, ${(E}_{z}^{i})$ represents the incident field column vectors of the N-element, and $\left(E_{z}^{s}\right)$ represents the scattered field column vectors of the M-element. M is the number of measurement points. $\left[G_{1}\right]$ is an N×N square matrix, while $\left[G_{2}\right]$ is an M×N matrix. $\left[\tau_{z}\right]$ is the diagonal matrix that is constituted by the dielectric coefficient: ${\left(\tau_{z}\right)}_{nn}=\varepsilon_{1}\left(x,y\right)-1$. $\left[I\right]$ is the N×N unit matrix.

#### 2.1.2. TE (Transverse Electric) Waves

TE electromagnetic waves are irradiated in the region S. $E_{x}^{i}\left(x,y\right)$ and $E_{y}^{i}\left(x,y\right)$ are the incident fields with the equations shown in (9) and (10), respectively.

(9) $$E_{x}^{i}\left(x,y\right)=-\sin\xi e^{-jk_{0}\left(x\cos\xi +y\sin\xi \right)}$$

(10) $$E_{y}^{i}\left(x,y\right)=\cos\xi e^{-jk_{0}\left(x\cos\xi +y\sin\xi \right)}$$

We adopt the vector potential technique to offset the coupling effect of the incident field ${\bar{E}}^{i}\left(x,y\right)=E_{x}^{i}\left(x,y\right)\hat{x}+E_{y}^{i}\left(x,y\right)\hat{y}$. The total electric field $\bar{E}\left(x,y\right)=E_{x}\left(x,y\right)\hat{x}+E_{y}\left(x,y\right)\hat{y}$ and the external scattered field ${\bar{E}}^{s}\left(x,y\right)=E_{x}^{s}\left(x,y\right)\hat{x}+E_{y}^{s}\left(x,y\right)\hat{y}$ are shown in Equations (11)–(14), respectively.

(11) $$\begin{array}{l} E_{x}^{\;}\left(x,y\right)=\left(\frac{{𝜕}^{2}}{𝜕x^{2}}+{k_{0}}^{2}\right)\left[\int_{s}^{\;}G\left(x,y;x\prime ,y\prime \right)\left(\varepsilon_{2}\left(x\prime ,y\prime \right)-1\right)E_{x}\left(x\prime ,y\prime \right)ds^{\prime }\right]\\ \;\;\;\;+\;\frac{{𝜕}^{2}}{𝜕x𝜕y}\left[\int_{s}^{\;}G\left(x,y;x\prime ,y\prime \right)\left(\varepsilon_{3}\left(x\prime ,y\prime \right)-1\right)E_{y}\left(x\prime ,y\prime \right)ds^{\prime }\right]\\ \;\;\;\;+{\overset{⃑}{E}}_{x}^{i}\left(x\prime ,y\prime \right) \end{array}$$

(12) $$\begin{array}{l} E_{y}^{\;}\left(x,y\right)=\frac{{𝜕}^{2}}{𝜕x𝜕y}\left[\int_{s}^{\;}G\left(x,y;x\prime ,y\prime \right)\left(\varepsilon_{2}\left(x\prime ,y\prime \right)-1\right)E_{x}\left(x\prime ,y\prime \right)ds^{\prime }\right]\\ \;\;\;\;+\left(\frac{{𝜕}^{2}}{𝜕y^{2}}+{k_{0}}^{2}\right)\left[\int_{s}^{\;}G\left(x,y;x\prime ,y\prime \right)\left(\varepsilon_{3}\left(x\prime ,y\prime \right)-1\right)E_{y}\left(x\prime ,y\prime \right)ds^{\prime }\right]\\ \;\;\;\;+{\overset{⃑}{E}}_{y}^{i}\left(x\prime ,y\prime \right) \end{array}$$

(13) $$\begin{array}{l} E_{x}^{s}\left(x,y\right)=\left(\frac{{𝜕}^{2}}{𝜕x^{2}}+{k_{0}}^{2}\right)\left[\int_{s}^{\;}G\left(x,y;x\prime ,y\prime \right)\left(\varepsilon_{2}\left(x\prime ,y\prime \right)-1\right)E_{x}\left(x\prime ,y\prime \right)ds^{\prime }\right]\\ \;\;\;\;+\frac{{𝜕}^{2}}{𝜕x𝜕y}\left[\int_{s}^{\;}G\left(x,y;x\prime ,y\prime \right)\left(\varepsilon_{3}\left(x\prime ,y\prime \right)-1\right)E_{y}\left(x\prime ,y\prime \right)ds^{\prime }\right] \end{array}$$

(14) $$\begin{array}{l} E_{y}^{s}\left(x,y\right)=\frac{{𝜕}^{2}}{𝜕x𝜕y}\left[\int_{s}^{\;}G\left(x,y;x\prime ,y\prime \right)\left(\varepsilon_{2}\left(x\prime ,y\prime \right)-1\right)E_{x}\left(x\prime ,y\prime \right)ds^{\prime }\right]\left(\frac{{𝜕}^{2}}{𝜕{y\;}^{2}}+{k_{0}}^{2}\right)\\ \;\;\;\;\left[\int_{s}^{\;}G\left(x,y;x\prime ,y\prime \right)\left(\varepsilon_{3}\left(x\prime ,y\prime \right)-1\right)E_{y}\left(x\prime ,y\prime \right)ds^{\prime }\right] \end{array}$$

If the permittivity tensor of the scatterer is known, the total electric field for (3), (11), and (12) can be solved first. The scattered field outside the scatterer can be obtained from (4), (13). (13), and (14) and can be converted to matrix equations as shown in (15) and (16), respectively,

(15) $$\left(\begin{array}{c} {-E}_{x}^{i}\\ {-E}_{y}^{i} \end{array}\right)=\left\{\left[\begin{array}{cc} \left[G_{3}\right] & \left[G_{4}\right]\\ \left[G_{4}\right] & \left[G_{5}\right] \end{array}\right]\left[\begin{array}{cc} \left[\tau_{x}\right] & 0\\ 0 & \left[\tau_{y}\right] \end{array}\right]-\left[\begin{array}{cc} \left[I\right] & 0\\ 0 & \left[I\right] \end{array}\right]\right\}\left(\begin{array}{c} E_{x}^{\;}\\ E_{y}^{\;} \end{array}\right)$$

(16) $$\left(\begin{array}{c} E_{x}^{s}\\ E_{y}^{s} \end{array}\right)=\left\{\left[\begin{array}{cc} \left[G_{6}\right] & \left[G_{7}\right]\\ \left[G_{7}\right] & \left[G_{8}\right] \end{array}\right]\left[\begin{array}{cc} \left[\tau_{x}\right] & 0\\ 0 & \left[\tau_{y}\right] \end{array}\right]\right\}\left(\begin{array}{c} E_{x}^{\;}\\ E_{y}^{\;} \end{array}\right)$$

and the Green’s function matrices are shown in (17)–(22)

(17) $$\left(G_{3}\right)=\left\{\begin{array}{c} -\frac{j\pi a_{n}J_{1}\left(k_{0}a_{n}\right)}{2{\rho^{3}}_{mn}}\times \left[k\rho_{mn}{\left(y_{m}-y_{n}\right)}^{2}{H_{0}}^{\left(2\right)}\left(k_{0}\rho_{mn}\right)\right.+\begin{array}{cc} \left.\left({\left(x_{m}-x_{n}\right)}^{2}-{\left(y_{m}-y_{n}\right)}^{2}\right){H_{1}}^{\left(2\right)}\left(k_{0}\rho_{mn}\right)\right] & ,m\neq n \end{array}\\ -\frac{j}{4}\left[\pi k_{0}a_{n}{H_{1}}^{\left(2\right)}\left(k_{0}a_{n}\right)-4j\right],\;m=n \end{array}\right.$$

(18) $$\left(G_{4}\right)=\left\{\begin{array}{c} -\frac{j\pi a_{n}J_{1}\left(k_{0}a_{n}\right)}{2{\rho^{3}}_{mn}}\left(x_{m}-x_{n}\right)\left(y_{m}-y_{n}\right)\times \begin{array}{cc} \left[2{H_{1}}^{\left(2\right)}\left(k_{0}\rho_{mn}\right)-k_{0}\rho_{mn}{H_{0}}^{\left(2\right)}\left(k_{0}\rho_{mn}\right)\right] & ,\;m\neq n\; \end{array}\\ 0,\;m=n \end{array}\right.$$

(19) $$\left(G_{5}\right)=\left\{\begin{array}{c} -\frac{j\pi a_{n}J_{1}\left(k_{0}a_{n}\right)}{2{\rho^{3}}_{mn}}\times \left[k_{0}\rho_{mn}{\left(x_{m}-x_{n}\right)}^{2}{H_{0}}^{\left(2\right)}\left(k_{0}\rho_{mn}\right)+\begin{array}{cc} \left.\left({\left(y_{m}-y_{n}\right)}^{2}-{\left(x_{m}-x_{n}\right)}^{2}\right)\right] & ,\;m\neq n\; \end{array}\right.\\ -\frac{j}{4}\left[\pi k_{0}a_{n}{H_{1}}^{\left(2\right)}\left(k_{0}a_{n}\right)-4j\right],\;m=n \end{array}\right.$$

(20) $$\left(G_{6}\right)=-\frac{j\pi a_{n}J_{1}\left(k_{0}a_{n}\right)}{2{\rho^{3}}_{mn}}\times \left[k_{0}\rho_{mn}{\left(y_{m}-y_{n}\right)}^{2}{H_{0}}^{\left(2\right)}\left(k_{0}\rho_{mn}\right)+\left({\left(x_{m}-x_{n}\right)}^{2}-{\left(y_{m}-y_{n}\right)}^{2}\right){H_{1}}^{\left(2\right)}\left(k_{0}\rho_{mn}\right)\right]$$

(21) $$\left(G_{7}\right)=-\frac{j\pi a_{n}J_{1}\left(k_{0}a_{n}\right)}{2{\rho^{3}}_{mn}}\left(x_{m}-x_{n}\right)\left(y_{m}-y_{n}\right)\left[2{H_{1}}^{\left(2\right)}\left(k_{0}\rho_{mn}\right)-k_{0}\rho_{mn}{H_{0}}^{\left(2\right)}\left(k_{0}\rho_{mn}\right)\right]$$

(22) $$\left(G_{8}\right)=-\frac{j\pi a_{n}J_{1}\left(k_{0}a_{n}\right)}{2{\rho^{3}}_{mn}}\left[k_{0}\rho_{mn}{\left(x_{m}-x_{n}\right)}^{2}{H_{0}}^{\left(2\right)}\left(k_{0}\rho_{mn}\right)+\left({\left(y_{m}-y_{n}\right)}^{2}-{\left(x_{m}-x_{n}\right)}^{2}\right){H_{1}}^{\left(2\right)}\left(k_{0}\rho_{mn}\right)\right]$$

where $\rho_{mn}=\sqrt{{\left(x_{m}-x_{n}\right)}^{2}+{\left(y_{m}-y_{n}\right)}^{2}}$ and $H_{0}^{\left(2\right)}$ is the second kind of the zero-order Hankel function. ${H_{1}}^{\left(2\right)}$ is the second kind of the first-order Hankel function, $J_{1}$ is the Bessel function of the first order. ($x_{n},y_{n}$) is the n-th source point and ($x_{m},y_{m}$) is the m-th observation point.

$\left(E_{x}^{\;}\right)$ and $\left(E_{y}^{\;}\right)$ represent the total electric field column vectors of the N-element, ${(E}_{x}^{i})and{(E}_{y}^{i})$ represent the incident field column vectors of the N-element, and $\left(E_{x}^{s}\right)and(E_{y}^{s})$ represent the scattered field column vectors of the M-element. M is the number of measurement points. $\left[G_{3}\right],\left[G_{4}\right],$ and $\left[G_{5}\right]$ are the N×N square matrices, while $\left[G_{6}\right]$, $\left[G_{7}\right],$ and $\left[G_{8}\right]$ are the M×N matrices. $\left[\tau_{x}\right]and\left[\tau_{y}\right]$ are the diagonal matrices with the dielectric coefficients of ${\left(\tau_{x}\right)}_{nn}=\varepsilon_{2}\left(\bar{r}\right)-1$ and ${\left(\tau_{y}\right)}_{nn}=\varepsilon_{3}\left(\bar{r}\right)-1$. $\left[I\right]$ is an N × N unit matrix.

### 2.2. Inverse Problem

#### Backpropagation Scheme (BPS)

We utilize the measured scattered field to estimate the dielectric constant distribution of anisotropic objects by the backpropagation scheme (BPS) to reduce the training difficulty of U-Net. Firstly, the backpropagation field is assumed to be proportional to the induced current $I_{z}^{b},I_{x}^{b}$, and $I_{y}^{b}$, as shown in (23) and (24).

(23) $$(I_{z}^{b})=\Upsilon_{m}\cdot {\left[G_{2}\right]}^{H}\left(E_{z}^{s}\right)$$

(24) $$\left(\begin{array}{c} I_{x}^{b}\\ I_{y}^{b} \end{array}\right)=\Upsilon_{e}\cdot {\left[\begin{array}{cc} \left[G_{6}\right] & \left[G_{7}\right]\\ \left[G_{7}\right] & \left[G_{8}\right] \end{array}\right]}^{H}\left(\begin{array}{c} E_{x}^{s}\\ E_{y}^{s} \end{array}\right)$$

where H stands for conjugate transpose.

According to (6), the loss function of the TM wave can be defined as:

(25) $$L_{m}^{b}(\Upsilon )={\left\|\left(E_{z}^{s}\right)-\left[G_{2}\right]\cdot \Upsilon_{m}\cdot {\left[G_{2}\right]}^{H}\left(E_{z}^{s}\right)\right\|}^{2}$$

According to Equation (16), the loss function of the TE wave can be defined as:

(26) $$L_{e}^{b}(\Upsilon )={\left\|\left(\begin{array}{c} E_{x}^{s}\\ E_{y}^{s} \end{array}\;\right)-\left[\begin{array}{cc} \left[G_{6}\right] & \left[G_{7}\right]\\ \left[G_{7}\right] & \left[G_{8}\right] \end{array}\right]\cdot \Upsilon_{e}\cdot {\left[\begin{array}{cc} \left[G_{6}\right] & \left[G_{7}\right]\\ \left[G_{7}\right] & \left[G_{8}\right] \end{array}\right]}^{H}\left(\begin{array}{c} E_{x}^{s}\\ E_{y}^{s} \end{array}\right)\right\|}^{2}$$

To minimize the loss function, the derivative must be zero. The analytical solutions for $\Upsilon_{m}$ and $\Upsilon_{e}$ are shown in (27) and (28), respectively,

(27) $$\Upsilon_{m}=\frac{{\left(E_{z}^{s}\;\right)}^{T}\cdot {\left(\left[G_{2}\right]\left({\left[G_{2}\right]}^{H}\cdot \left(E_{z}^{s}\right)\right)\right)}^{*}}{{\left\|\left[G_{2}\right]\left({\left[G_{2}\right]}^{H}\cdot \left(E_{z}^{s}\right)\right)\right\|}^{2}}$$

(28) $$\Upsilon_{e}=\frac{{\left(\begin{array}{c} E_{x}^{s}\\ E_{y}^{s} \end{array}\;\right)}^{T}\cdot {\left(\left[\begin{array}{cc} \left[G_{6}\right] & \left[G_{7}\right]\\ \left[G_{7}\right] & \left[G_{8}\right] \end{array}\right]\left({\left[\begin{array}{cc} \left[G_{6}\right] & \left[G_{7}\right]\\ \left[G_{7}\right] & \left[G_{8}\right] \end{array}\right]}^{H}\cdot \left(\begin{array}{c} E_{x}^{s}\\ E_{y}^{s} \end{array}\right)\right)\right)}^{*}}{{\left\|\left[\begin{array}{cc} \left[G_{6}\right] & \left[G_{7}\right]\\ \left[G_{7}\right] & \left[G_{8}\right] \end{array}\right]\left({\left[\begin{array}{cc} \left[G_{6}\right] & \left[G_{7}\right]\\ \left[G_{7}\right] & \left[G_{8}\right] \end{array}\right]}^{H}\cdot \left(\begin{array}{c} E_{x}^{s}\\ E_{y}^{s} \end{array}\right)\right)\right\|}^{2}}$$

where T stands for transpose and * stands for complex conjugate.

Once $\Upsilon_{m}$ is known, (23) is used to find the induced current of the TM wave. And the backpropagation total field $E_{z}^{b}$ is solved by (5) to produce (29).

(29) $$\left(E_{z}^{b}\right)=\left(E_{z}^{i}\right)+\left[G_{1}\right]\left(I_{z}^{b}\right)$$

Similarly, (24) can be used to find the induced current of the TE wave. The backpropagation total field $E_{x}^{b}$ and $E_{y}^{b}$ are solved by (15) to produce (30).

(30) $$\left(\begin{array}{c} E_{x}^{b}\\ E_{y}^{b} \end{array}\right)=\left(\begin{array}{c} E_{x}^{i}\\ E_{y}^{i} \end{array}\right)+\left[\begin{array}{cc} \left[G_{3}\right] & \left[G_{4}\right]\\ \left[G_{4}\right] & \left[G_{5}\right] \end{array}\right]\left(\begin{array}{c} I_{x}^{b}\\ I_{y}^{b} \end{array}\right)$$

The relationship between the induced current $I_{z}^{b}$ and the contrast $\left[\tau_{z}^{b}\right]$ is shown in (31).

(31) $$\left(I_{z,p}^{b}\right)=diag\left(\left[\tau_{z}^{b}\right]\right)\left(E_{z}^{b}\right)$$

The relationship between the induced current $I_{x,p}^{b}$ and $I_{y,p}^{b}$ with contrasts $\left[\tau_{x}^{b}\right]$ and $\left[\tau_{y}^{b}\right]$ is shown in (32).

(32) $$\left(\begin{array}{c} I_{x,p}^{b}\\ I_{y,p}^{b} \end{array}\right)=diag\left(\left[\begin{array}{cc} \left[\tau_{x}^{b}\right] & 0\\ 0 & \left[\tau_{y}^{b}\right] \end{array}\right]\right)\left(\begin{array}{c} E_{x}^{b}\\ E_{y}^{b} \end{array}\right)$$

where p represents each incidence and $\left[\tau_{z}^{b}\right],\left[\tau_{x}^{b}\right],and\left[\tau_{y}^{b}\right]$ represent the dielectric constant profiles of the backpropagation.

The least squares method is used to calculate all incidences of (31) to obtain the contrast $\left[\tau_{z}^{b}\right]$ of the n-th element, as shown in (33).

(33) $$\left[\tau_{z}^{b}\right]=\frac{\sum_{p=1}^{Ni}I_{z,\;p}^{b}\left(n\right)\cdot {\left[E_{z}^{b}\left(n\right)\right]}^{*}}{\sum_{p=1}^{Ni}{\left\|E_{z}^{b}\left(n\right)\right\|}^{2}}$$

Similarly, the contrasts $\left[\tau_{x}^{b}\right]$ and $\left[\tau_{y}^{b}\right]$ of the n-th element can be obtained by calculating all incidences of (32) using the least squares method, as shown in (34)

(34) $$\left[\begin{array}{c} \tau_{x}^{b}\\ \tau_{y}^{b} \end{array}\right]=\sum_{p=1}^{Ni}\frac{\begin{array}{c} I_{x,p}^{b}\left(n\right)\\ I_{y,p}^{b}\left(n\right) \end{array}\cdot {\left[\left(\begin{array}{c} E_{x}^{b}\\ E_{y}^{b} \end{array}\right)\left(n\right)\right]}^{*}}{{\left\|\begin{array}{c} E_{x}^{b}\left(n\right)\\ E_{y}^{b}\left(n\right) \end{array}\right\|}^{2}}$$

where *p* represents the number of incident waves.

## 3. Convolutional Neural Network

A CNN is a machine learning model inspired by the biological nervous system, which simulates the information processing and transfers among neurons in the brain. The applications cover a wide range, such as graphical image recognition, natural language processing, computer vision, speech recognition, and so on. The advantage of convolutional neural network is that the system is capable of changing its internal structure based on external information by its adaptive characteristic for learning.

The CNN architecture includes a convolutional layer, a pooling layer, a ReLu layer, and a fully connected layer. The convolutional layer detects features in an input image by converting it into a matrix. It slides various kernels over the matrix, convolving local regions with the kernels to generate feature maps. The kernels are the important weight parameters in the convolutional layer. They are used to detect features such as edges, colors, textures, etc., by the size and step of each slide. The pooling layer is used to reduce the spatial dimension of the feature mapping and the size of the feature image, which in turn speeds up computation and avoids the occurrence of over-fitting. The ReLu layer is used to quell the non-linear features to improve the training effectiveness of the CNN and to mitigate the problem of gradient vanishing. It also possesses sparse activation to promote the generalization ability of CNN models. The fully connected layer is used to expand the feature matrix into a one-dimensional vector.

In this paper, U-Net, a network architecture for image segmentation in a CNN, is applied as the training model. Its feature extraction step is more complicated than the CNN, which consists of two parts, an encoder and a decoder. The advantages of U-Net include the following:

(1) U-Net has a strong generative capability even with limited training data.
(2) Its skip-connect method can effectively mitigate the problem of gradient vanishing during the training process.
(3) Through U-Net’s down-sampling network, the perceptual range is expanded, thereby enhancing the accuracy of pixel predictions.
(4) The batch normalization layers expedite training and reduce gradient dependency on parameters or initial values.

**Figure 2:**
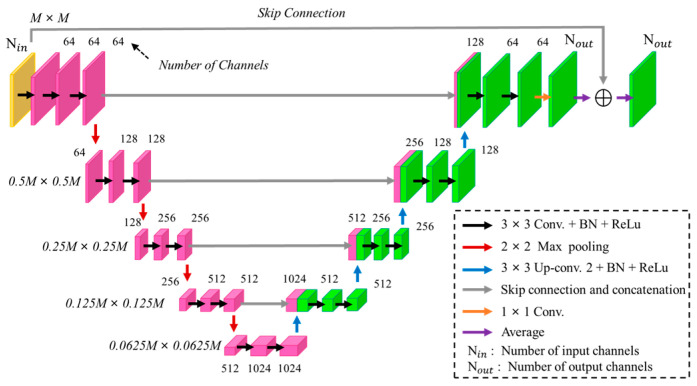
U-Net architecture.

The U-Net architecture in Figure 2 consists of a right half extended network and a left half contracted network. The left half includes overlapping 3 × 3 convolutional layers, batch normalization layers, and ReLU activation layers. The pooling layer in the contracted and extended networks are a 2 × 2 max-pooling layer and a 3 × 3 transposed convolutional layer, respectively. The fully connected layer is a 1 × 1 convolutional layer. The number of incidences $N_{i}$ is equal to the number of output channels $N_{out}$. By averaging the outputs of the fully connected layer and inputting them into the regression layer, the error value of the dielectric coefficient distribution can be calculated.

The minimization equation for the TM wave dielectric coefficient $\varepsilon_{z}$ is shown in (35)

(35) $$\begin{array}{c} argmin\\ A_{i1},i \end{array}:\sum_{N=1}^{N_{t}}f\left(A_{i1}\left(\varepsilon_{1}^{\alpha }\right),\;\varepsilon_{1}\right)+Q_{1}\left(i\right)$$

And the minimization equation for the TE wave dielectric coefficients $\varepsilon_{x}$ and $\varepsilon_{y}$ is shown in (36)

(36) $$\begin{array}{c} argmin\\ A_{i2},i \end{array}:\sum_{N=1}^{N_{t}}f\left(A_{i2}\left(\begin{array}{c} \varepsilon_{2}^{\alpha }\\ \varepsilon_{3}^{\alpha } \end{array}\right),\;\left(\begin{array}{c} \varepsilon_{2}\\ \varepsilon_{3} \end{array}\right)\right)+Q_{2}\left(i\right)$$

where $A_{i1}$ and $A_{i2}$ represent the neural network structure, i is the parameter of the neural network, f is the error, $\varepsilon_{1}^{\alpha }$, $\varepsilon_{2}^{\alpha }$, and $\varepsilon_{3}^{\alpha }$ are the approximate dielectric coefficients, and $Q_{1}\left(i\right)$ and $Q_{2}\left(i\right)$ are the regularization functions.

The deep learning training method implemented in this paper is adaptive moment estimation (ADAM), which is a first-order gradient optimization algorithm for stochastic objective functions based on low-order moment adaptive estimation. Its strengths include easy implementation, high computational efficiency, low memory capacity requirements, and constant diagonal rescaling of the gradient. This method also provides an apt solution for non-stationary and highly noisy or sparse gradient problems.

## 4. Numerical Results

The aim of this research is to reconstruct the electromagnetic imaging of free-space anisotropic objects in the simulation environment, as shown in Figure 1. The incident wave frequency is 2, 3, and 4 GHz, and the edge length of the scatterer is subdivided into sizes smaller than $\frac{0.2\lambda_{0}}{\sqrt{\varepsilon_{s}}}$, where $\lambda_{0}$ represents the free-space wavelength, and $\varepsilon_{s}$ represents the maximum relative permittivity of the anisotropic scatterers. In the simulation environment, separate TE and TM waves are emitted to illuminate the anisotropic objects. We deploy 32 transmitters and 32 receivers for measurements, with 5% and 20% Gaussian noise added, respectively. In the TM polarization process, the transmitters and receivers are placed 11.25 degrees apart. However, since the magnitudes of the incident fields $E_{x}^{i}$ and $E_{y}^{i}$ are affected by the incident angles during the reconstruction process of TE waves, we carefully select the incident angle of TE polarization. In the training, BPS is used to estimate the dielectric constant profiles of the anisotropic objects. The data are further divided into 50% for training, 25% for validating, and 25% for testing. ADAM deep learning method is used to train U-Net. The initial learning rate and the max epoch are set at $10^{-3}$ and 40, respectively. The 0.1 gradient descent is run for every 20 epochs, and the batch size is 32; the gradient decay factor and the squared gradient decay factor are assumed as 0.9 and 0.99, respectively.

In order to evaluate the performance of the reconstruction results, we define the root mean squared error (RMSE) as shown in (37)

(37) $$RMSE=\frac{1}{M_{T}}\sum_{i=1}^{M_{T}}\frac{{\left\|I-I^{\alpha }\right\|}_{F}}{{\left\|I\right\|}_{F}}$$

where I and $I^{\alpha }$ represent the original image and reconstructed image, respectively, $M_{T}$ represents the total number of tests, and F represents the Frobenius norm.

The structural similarity index measure (SSIM) in (38) is employed to compare the dissimilarity of the reconstructed results

(38) $$SSIM=\frac{\left(2\mu_{\tilde{y}}\mu_{y}+C_{1}\right)\left(2\sigma_{\tilde{y}y}+C_{2}\right)}{\left(\mu_{\tilde{y}}^{2}+\mu_{y}^{2}+C_{1}\right)\left(\sigma_{\tilde{y}}^{2}+\sigma_{y}^{2}+C_{2}\right)}$$

where $\tilde{y}$ and y represent the reconstructed image and the original image, respectively, the pixel sample mean of y is $\mu_{y}$, the variance of y is $\sigma_{y}^{2}$, and the covariance of $\tilde{y}$ and y is $\sigma_{\tilde{y}y}$. $C_{1}$ and $C_{2}$ are two variables used to stabilize the division with a weak denominator.

The improved loss function used in this proposal is formed by combining SSIM with RMSE as shown in (39).

(39) $$L_{full}\left(\hat{y},y\right)=\sum_{j=1}^{N_{p}}L_{RMSE}\left({\hat{y}}_{j},y_{j}\right)+\alpha L_{SSIM}\left({\hat{y}}_{j},y_{j}\right)$$

where ${\hat{y}}_{j}$ and $y_{j}$ represent the j-th pixel of image $\hat{y}$ and y, respectively, and $L_{SSIM}$ and $L_{RMSE}$ represent the loss function value of SSIM and RMSE, respectively. α is set at 0.5, representing the weight of SSIM.

### 4.1. Relative Permittivity Ranging from 1 to 1.5

In this study, we establish dielectric constant profiles within the interval of 1.0 to 1.5. We assume that there are 10 scatterers of different dielectric constant profiles situated randomly at 80 various locations with 20% random Gaussian noise added. The dielectric constant distribution is derived from the multifrequency scattered field data employing BPS, and these data are input into a CNN for electromagnetic image reconstruction. Subsequently, we compare the reconstruction results obtained from both the single-frequency and multifrequency methodologies.

**Figure 3:**
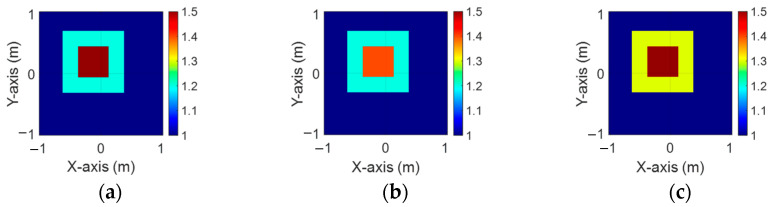
Ground truth: (**a**) $\varepsilon_{1}$, (**b**) $\varepsilon_{2}$, and (**c**) $\varepsilon_{3}$ for Case 4.1.

**Figure 4:**
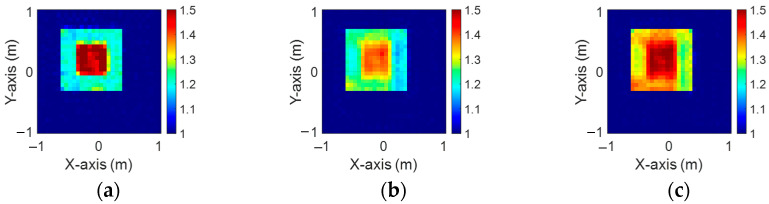
Image reconstructed using BPS with 2 GHz input: (**a**) $\varepsilon_{1}$, (**b**) $\varepsilon_{2}$, and (**c**) $\varepsilon_{3}$ for Case 4.1.

**Figure 5:**
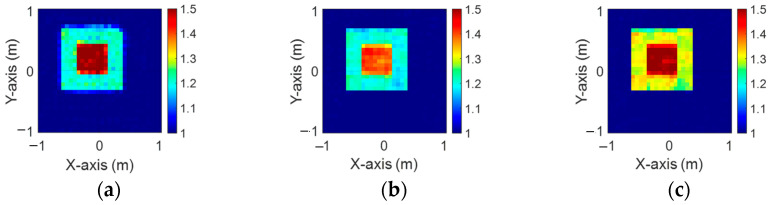
Image reconstructed using BPS with 3 GHz input: (**a**) $\varepsilon_{1}$, (**b**) $\varepsilon_{2}$, and (**c**) $\varepsilon_{3}$ for Case 4.1.

**Figure 6:**
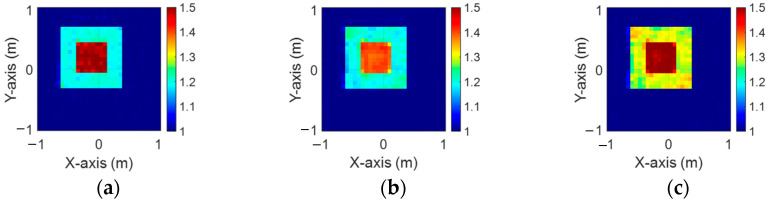
Image reconstructed using BPS with 4 GHz input: (**a**) $\varepsilon_{1}$, (**b**) $\varepsilon_{2}$, and (**c**) $\varepsilon_{3}$ for Case 4.1.

**Figure 7:**
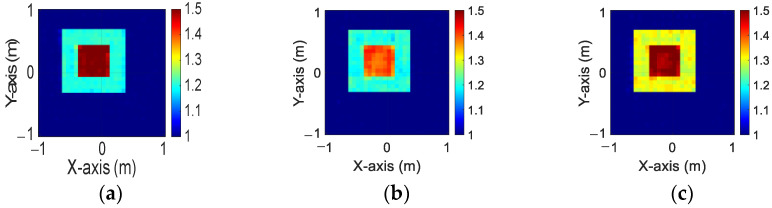
Image reconstructed using BPS with multifrequency input: (**a**) $\varepsilon_{1}$, (**b**) $\varepsilon_{2}$, and (**c**) $\varepsilon_{3}$ for Case 4.1.

Figure 3a–c illustrate the initial dielectric constant profiles of the $\varepsilon_{1}$, $\varepsilon_{2}$, and $\varepsilon_{3}$ scatterers, respectively. Figure 4a–c illustrate, respectively, the reconstructed dielectric constant profiles of $\varepsilon_{1}$, $\varepsilon_{2}$, and $\varepsilon_{3}$ by the CNN with 2 GHz input to BPS. Figure 5a–c illustrate, respectively, the reconstructed dielectric constant profiles of $\varepsilon_{1}$, $\varepsilon_{2}$, and $\varepsilon_{3}$ by the CNN with 3 GHz input to BPS. Figure 6a–c illustrate, respectively, the reconstructed dielectric constant profiles of $\varepsilon_{1}$, $\varepsilon_{2}$, and $\varepsilon_{3}$ by the CNN with 4 GHz input to BPS. Figure 7a–c illustrate, respectively, the reconstructed dielectric constant profiles of $\varepsilon_{1}$, $\varepsilon_{2}$, and $\varepsilon_{3}$ by the CNN with multifrequency input to BPS. The results indicate that multifrequency outperforms the single frequency in reconstructing the dielectric coefficient distribution. Table 1 presents the SSIM and RMSE values of the reconstruction results. We can see that the reconstructed images using multifrequency data demonstrate superior error rates and similarity compared with those using single-frequency data in both the TE and TM cases.

### 4.2. Relative Permittivity Ranging from 1.5 to 2

In this case, we configure the dielectric constant distribution ranging from 1.5 to 2. We consider 10 scatterers, each with varying dielectric constants, that can move freely among 80 different positions within the measurement area. To simulate realistic conditions, we introduce 5% Gaussian noise. We initially estimate the dielectric constant distribution from multifrequency scattered field data via BPS, which is then input into the CNN for electromagnetic image reconstruction. Eventually, we compare the reconstruction results obtained from the single-frequency and multifrequency methods.

**Figure 8:**
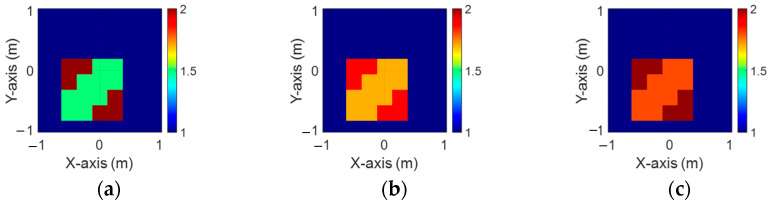
Ground truth: (**a**) $\varepsilon_{1}$, (**b**) $\varepsilon_{2}$, and (**c**) $\varepsilon_{3}$ for Case 4.2.

**Figure 9:**
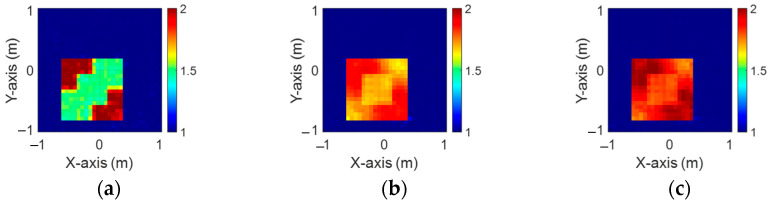
Image reconstructed using BPS with 2 GHz input: (**a**) $\varepsilon_{1}$, (**b**) $\varepsilon_{2}$, and (**c**) $\varepsilon_{3}$ for Case 4.2.

**Figure 10:**
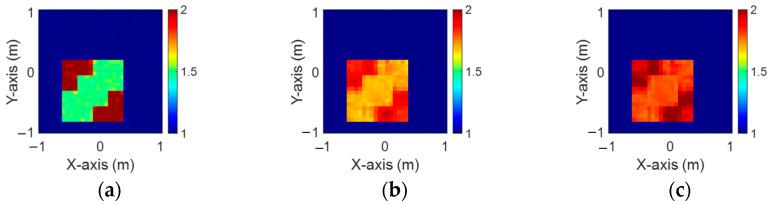
Image reconstructed using BPS with 3 GHz input: (**a**) $\varepsilon_{1}$, (**b**) $\varepsilon_{2}$, and (**c**) $\varepsilon_{3}$ for Case 4.2.

**Figure 11:**
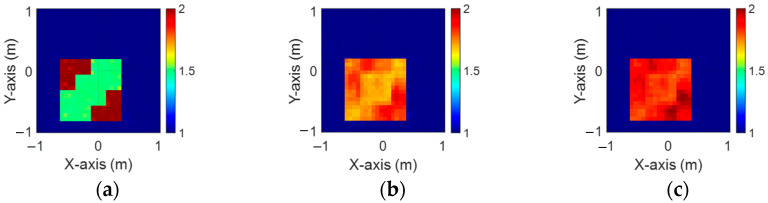
Image reconstructed using BPS with 4 GHz input: (**a**) $\varepsilon_{1}$, (**b**) $\varepsilon_{2}$, and (**c**) $\varepsilon_{3}$ for Case 4.2.

**Figure 12:**
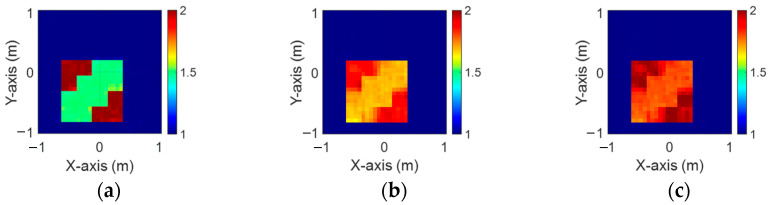
Image reconstructed using BPS with multifrequency input: (**a**) $\varepsilon_{1}$, (**b**) $\varepsilon_{2}$, and (**c**) $\varepsilon_{3}$ for Case 4.2.

Figure 8a–c depict the initial dielectric constant profiles of the $\varepsilon_{1}$, $\varepsilon_{2}$, and $\varepsilon_{3}$ scatterers, respectively. Figure 9a–c show the reconstructed dielectric constant profiles for $\varepsilon_{1}$, $\varepsilon_{2}$, and $\varepsilon_{3}$ by the CNN using BPS with 2 GHz input. Figure 10a–c and Figure 11a–c present, respectively, the reconstructed dielectric constant profiles for $\varepsilon_{1}$, $\varepsilon_{2}$, and $\varepsilon_{3}$ using the CNN with BPS for 3 GHz and 4 GHz inputs. Figure 12a–c present the reconstructed dielectric constant profiles for $\varepsilon_{1}$, $\varepsilon_{2}$, and $\varepsilon_{3}$ by the CNN using BPS with multifrequency input. Figure 9, Figure 10, Figure 11 and Figure 12 demonstrate that BPS with 2 GHz, 3 GHz, 4 GHz, and multifrequency inputs can only reconstruct the scatterer positions and provide a coarse dielectric constant profile. The findings show that BPS using multifrequency data achieves a more precise reconstruction of the dielectric coefficient distribution compared with single-frequency data. Based on the SSIM and RMSE values shown in Table 2, it is clear that the images reconstructed using multifrequency data perform better than those reconstructed using single-frequency data in terms of both similarity and the error rate.

### 4.3. Relative Permittivity Ranging from 2 to 2.5

The Modified National Institute of Standards and Technology (MNIST) database is a prominent dataset for image recognition in machine learning and deep learning. It comprises 70,000 grayscale images of handwritten digits from 0 to 9, each measuring 28 × 28 pixels. In our study, we utilize the MNIST dataset with a dielectric distribution range of 2 to 2.5. We simulate 8000 randomized images, adding 5% Gaussian noise, to estimate the dielectric coefficient distribution. These data are processed using BPS on both single-frequency and multifrequency scattering fields and subsequently input into the CNN for electromagnetic image reconstruction. Finally, we compare the reconstruction results obtained from the two different input datasets.

**Figure 13:**
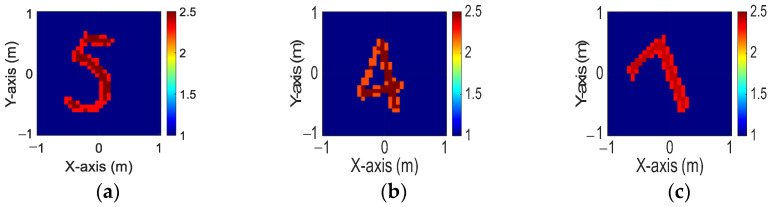
Ground truth: (**a**) $\varepsilon_{1}$, (**b**) $\varepsilon_{2}$, and (**c**) $\varepsilon_{3}$ for Case 4.3.

**Figure 14:**
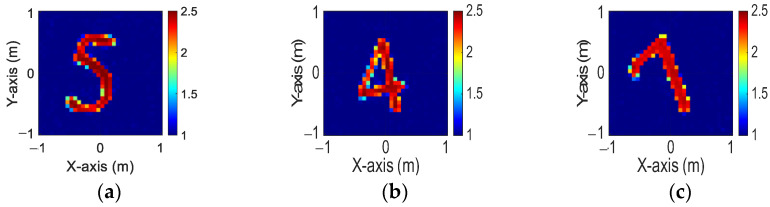
Image reconstructed using BPS with 2 GHz input: (**a**) $\varepsilon_{1}$, (b) $\varepsilon_{2}$, and (**c**) $\varepsilon_{3}$ for Case 4.3.

**Figure 15:**
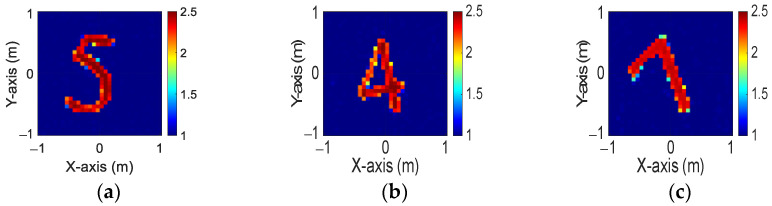
Image reconstructed using BPS with 3 GHz input: (**a**) $\varepsilon_{1}$, (**b**) $\varepsilon_{2}$, and (**c**) $\varepsilon_{3}$ for Case 4.3.

**Figure 16:**
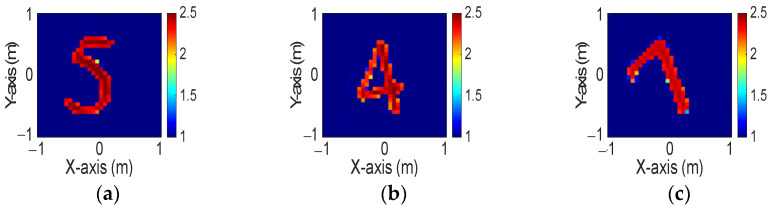
Image reconstructed using BPS with 4 GHz input: (**a**) $\varepsilon_{1}$, (**b**) $\varepsilon_{2}$, and (**c**) $\varepsilon_{3}$ for Case 4.3.

**Figure 17:**
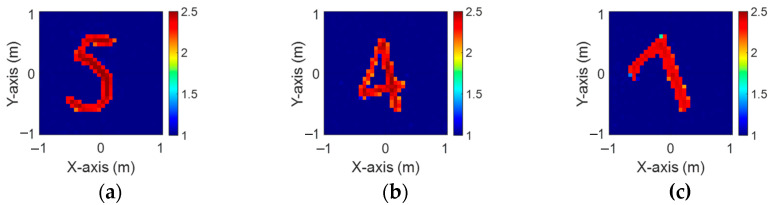
Image reconstructed using BPS with multifrequency input: (**a**) $\varepsilon_{1}$, (**b**) $\varepsilon_{2}$, and (**c**) $\varepsilon_{3}$ for Case 4.3.

Figure 13a–c display the initial dielectric constant profiles of the scatterers $\varepsilon_{1}$, $\varepsilon_{2}$, and $\varepsilon_{3}$, respectively. Figure 14a–c present the reconstructed dielectric constant profiles for $\varepsilon_{1}$, $\varepsilon_{2}$, and $\varepsilon_{3}$ using the CNN with BPS based on 2 GHz input. Similarly, Figure 15a–c and Figure 16a–c show the reconstructed dielectric constant profiles for $\varepsilon_{1}$, $\varepsilon_{2}$, and $\varepsilon_{3}$ using the CNN with BPS based on 3 GHz and 4 GHz inputs, respectively. Likewise, in cases A and B, these figures demonstrate that BPS with 2 GHz, 3 GHz, and 4 GHz inputs can only reconstruct the scatterer positions and provide a coarse dielectric constant profile. Figure 17a–c display the reconstructed dielectric constant profiles for $\varepsilon_{1}$, $\varepsilon_{2}$, and $\varepsilon_{3}$ using the CNN with BPS based on multifrequency input. The simulation outcomes also indicate that using multifrequency data yields a more accurate reconstruction of the dielectric coefficient distribution compared with single-frequency data. In Table 3, the SSIM and RMSE values for the reconstruction results confirm that images reconstructed with multifrequency data exhibit lower error rates and higher similarity compared with those reconstructed with single-frequency data.

### 4.4. Research Dataset

In this study, we utilize a dataset from the Fresnel Institute to validate the effectiveness of our proposed multifrequency BPS in both the TE and TM schemes. The research dataset is set up in a test environment with eight transmitters and 241 receivers. The transmitters are positioned 1.67 m from the object under test. As horn antennas are used to measure the scattered field, no transmitters are placed adjacent to the receivers. We use the FoamDielExt measurement data for both the TE and TM cases, comprising a small cylinder (Berlon) and a large cylinder (SAITEC SBF 300). The small cylinder has a dielectric constant of $\varepsilon_{r}=3\;\pm \;0.3$ and a diameter of 31 mm, while the diameter of the large cylinder is 80 mm with a dielectric constant of $\varepsilon_{r}=1.45\;\pm \;0.15$ [29].

**Figure 18:**
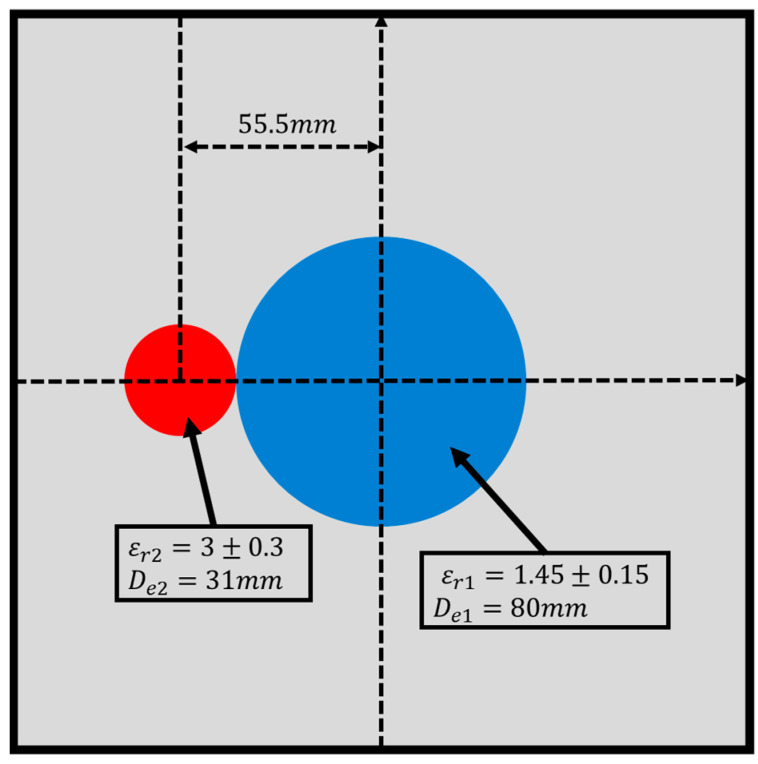
The schematic diagram of FoamDielExt.

In the simulation, the scatterer is placed in a 320 mm × 320 mm measurement area and illuminated with TE and TM waves at 2 GHz, 3 GHz, 4 GHz, and multifrequency. The received scattered fields are then calibrated. During calibration, we normalize using the scattered field received from the opposite side of the incident angle. The schematic diagram of FoamDielExt is shown in Figure 18.

**Figure 19:**
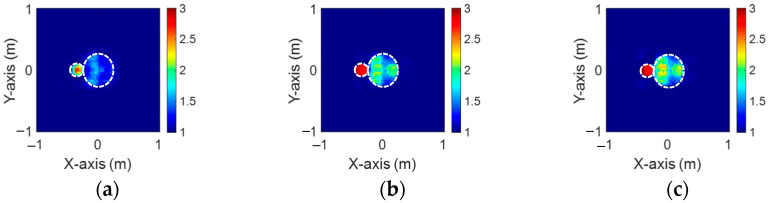
Image reconstructed using BPS with 2 GHz input: (**a**) $\varepsilon_{1}$, (**b**) $\varepsilon_{2}$, and (**c**) $\varepsilon_{3}$ for Case 4.4.

**Figure 20:**
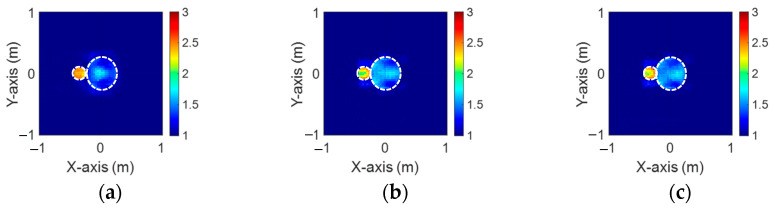
Image reconstructed using BPS with 3 GHz input: (**a**) $\varepsilon_{1}$, (**b**) $\varepsilon_{2}$, and (**c**) $\varepsilon_{3}$ for Case 4.4.

**Figure 21:**
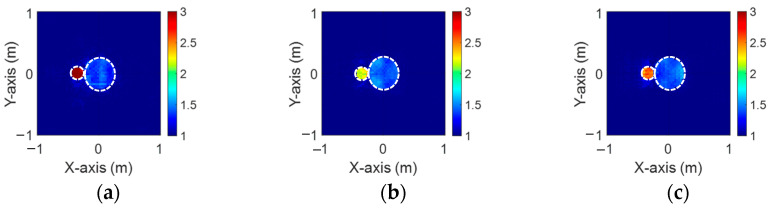
Image reconstructed using BPS with 4 GHz input: (**a**) $\varepsilon_{1}$, (**b**) $\varepsilon_{2}$, and (**c**) $\varepsilon_{3}$ for Case 4.4.

**Figure 22:**
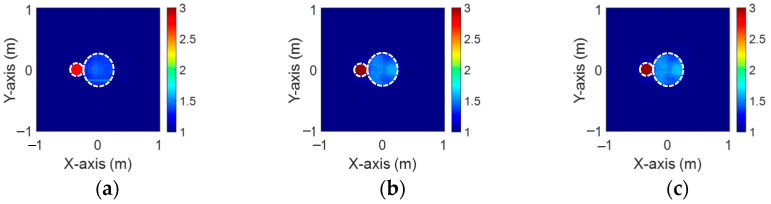
Image reconstructed using BPS with multifrequency input: (**a**) $\varepsilon_{1}$, (**b**) $\varepsilon_{2}$, and (**c**) $\varepsilon_{3}$ for Case 4.4.

Figure 19a–c, Figure 20a–c, and Figure 21a–c show the reconstructed dielectric constant profiles of $\varepsilon_{1}$, $\varepsilon_{2}$, and $\varepsilon_{3}$ using 2 GHz, 3 GHz, and 4 GHz input-based BPS, respectively. All these data reveal that the BPS based on 2 GHz, 3 GHz, and 4 GHz inputs are only able to reconstruct the position of the scatterer and provide a rough distribution of the dielectric constant. Figure 22a–c illustrate the reconstructed dielectric constant profiles of $\varepsilon_{1}$, $\varepsilon_{2}$, and $\varepsilon_{3}$ using the multifrequency input-based CNN and BPS. The simulation results show that multifrequency data from BPS are capable of reconstructing more accurate dielectric coefficients. Table 4 lists the SSIM and RMSE values of the reconstruction results for the research dataset.

## 5. Conclusions

The innovation of this paper lies in utilizing SSIM and RMSE to generate an improved loss function for reducing reconstruction artifacts. The numerical results indicate that an electromagnetic image can be significantly improved by the enhanced CNN model. Moreover, a comparison between single-frequency and multifrequency is also performed in terms of high accuracy and stability. By analyzing the anisotropic objects reconstructed by the TE and TM waves, we can see that despite the addition of 5% and 20% noises in the simulation environment, the images are still outstanding. Therefore, we conclude that through our proposed method of integrating BPS with appropriate CNN parameters, excellent reconstruction performance can be achieved for anisotropic objects located in free space under different dielectric coefficient distributions or different noise levels.

In addition, since a combination of SSIM and RMSE is used to optimize the loss function in this paper, other differentiable perceptual metrics may also be used to construct loss functions, which in turn inspires a new way of thinking for the current research. In the future, we intend to extend our exploration associated will anisotropic objects buried in half space or multi-layer space.

## Figures and Tables

**Table 1:** SSIM and RMSE for the permittivity range of 1–1.5 with 20% noise.

Reconstruction Performance		$\varepsilon_{1}$	$\varepsilon_{2}$	$\varepsilon_{3}$
2 GHz	SSIM	96.64%	95.9%	96.7%
	RMSE	2.19%	2.44%	2.37%
3 GHz	SSIM	96.16%	98.6%	98.7%
	RMSE	2.02%	1.15%	1.31%
4 GHz	SSIM	98.94%	98.7%	98.9%
	RMSE	1.12%	1.05%	1.17%
Multifrequency	SSIM	99.05%	99.01%	99.04%
	RMSE	0.83%	0.88%	0.93%

**Table 2:** SSIM and RMSE for the permittivity range of 1.5–2 with 5% noise.

Reconstruction Performance		$\varepsilon_{1}$	$\varepsilon_{2}$	$\varepsilon_{3}$
2 GHz	SSIM	97.9%	97.6%	97.7%
	RMSE	1.76%	2.03%	1.98%
3 GHz	SSIM	98.9%	98.5%	98.8%
	RMSE	1.1%	1.47%	1.43%
4 GHz	SSIM	99%	98.56%	97.2%
	RMSE	0.87%	2.59%	2.49%
Multifrequency	SSIM	99.33%	98.8%	98.8%
	RMSE	0.8%	1.38%	1.34%

**Table 3:** SSIM and RMSE for the permittivity range of 2–2.5 with 5% noise.

Reconstruction Performance		$\varepsilon_{1}$	$\varepsilon_{2}$	$\varepsilon_{3}$
2 GHz	SSIM	91.1%	93.81%	90.93%
	RMSE	9%	6.3%	6.8%
3 GHz	SSIM	96.11%	95.2%	92.9%
	RMSE	5.83%	3.37%	4.78%
4 GHz	SSIM	97.01%	97.07%	95.29%
	RMSE	5.23%	2.77%	4.42%
Multifrequency	SSIM	97.36%	97.62%	96.62%
	RMSE	5.02%	2.71%	2.48%

**Table 4:** SSIM and RMSE for the research dataset.

Reconstruction Performance		$\varepsilon_{1}$	$\varepsilon_{2}$	$\varepsilon_{3}$
2 GHz	SSIM	92.1%	92.96%	92.44%
	RMSE	9.68%	7.41%	8.98%
3 GHz	SSIM	93%	93.09%	94.04%
	RMSE	6.47%	7.29%	6.74%
4 GHz	SSIM	94.13%	95.16%	95.43%
	RMSE	3.78%	6.89%	4.97%
Multifrequency	SSIM	97.03%	97.35%	97.34%
	RMSE	3.4%	0.48%	0.53%

## Data Availability

Data are contained within the article.
